# Fulminant skin necrosis and massive deep venous thrombosis as a part of a clinical presentation of polyarteritis nodosa -case report

**DOI:** 10.1186/1546-0096-12-S1-P351

**Published:** 2014-09-17

**Authors:** Adisa Čengić, Senka Mesihović Dinarević, Aida Omerčahić Dizdarević, Velma Selmanović, Emir Kurtalić

**Affiliations:** 1 Allergology, Rheumatology and Clinical Immunology, University Clinical Center Sarajevo, Bosnia and Herzegovina; 2 Pediatric Cardiology, Childrens Hospital, University Clinical Center Sarajevo, Bosnia and Herzegovina; 3 Department for Adult Angiology, Hospital for Vascular Diseases, Sarajevo, Bosnia and Herzegovina

## Introduction

Polyarteritis Nodosa (PAN) is a systemic, necrotizing vasculitis that affects medium-sized and small muscular arteries resulting in microaneurysm formation. In the presteroid era, mortality was high and the diagnosis was exclusively made postmortem.

## Objectives

Our goal is to present very challenging, biopsy proven, case od PAN which resulted in massive necrotic skin lesions and deep venous thrombosis.

## Methods

We report the case of 8 year old boy who was referred to our hospital with 11 days history of high fever (39.5°C), fatigue, anorexia and weight loss. Two days prior to admission he began to complain of pain in the calves muscles, developed petechial skin rash and hematomas, gross haematuria and gastrointestinal (GI) bleeding. At admission was febrile, in very poor condition, hyperventilating, with calf muscle tenderness (mostly gastrocnemius and soleus muscles), scrotal pain, unable to walk. Skin findings: livedo reticularis, multiple hematomas, petechie, subcutaneous nodules. On the second day he developed cutaneous necrosis, five days after admission bilateral legs edema was observed. Doppler ultrasonography showed massive deep end superficial venous thrombosis. He was tested negative for hepatitis C antibodies, perinuclear antineutrophilic cytoplasmic antibody (pANCA) and cytoplasmic antineutrophilic cytoplasmic antibody (cANCA). A deep skin biopsy was performed and showed fibrinoid necrosis of small muscular arteries with leukocytic infiltrate; finding compatible with polyarteritis nodosa. Kidney biopsy was contraindicated because the boy had agenesis of the right kidney.

## Results

Patient responded well to the aggressive immunosuppressive therapy in terms of halting progression of skin necrosis and gradually resolving haematuria, GI bleeding, muscle and scrotal pain**.** Thrombosis were treated with intravenous and subcutaneous heparin with good respons. Cutaneous necrosis required skin transplantation. At present time, 18 months after disease onset, PAN is in remission and there are total recanalisation of deep and superficial lower extremities veins.

## Conclusion

PAN is life-threatening disease that often presents with multiorgan involment, but is rarely associated with deep venous thrombosis. Pain and swelling of the lower extremities is not always a symptom of the disease itself, but may indicate another very serious condition such as deep venous thrombosis. Disease requires promt diagnosis and aggressive immunosuppressive treatment to reduce morbidity and mortality.

## Disclosure of interest

None declared.

